# *Decatropis bicolor* (Zucc.) Radlk essential oil induces apoptosis of the MDA-MB-231 breast cancer cell line

**DOI:** 10.1186/s12906-016-1136-7

**Published:** 2016-08-05

**Authors:** C. C. Estanislao Gómez, A. Aquino Carreño, D. G. Pérez Ishiwara, E. San Martín Martínez, J. Morales López, N. Pérez Hernández, M. C. Gómez García

**Affiliations:** 1 Programa de Doctorado en Biotecnología, Escuela Nacional de Medicina y Homeopatía, Instituto Politécnico Nacional, Guillermo Massieu Helguera No. 239, Fracc. La Escalera, Ticomán, Delg. Gustavo A. Madero, Mexico, C.P. 07320 D. F. Mexico; 2 Departamento de Ciencias Químico Biológicas, Instituto de Ciencias Biomédicas, Universidad Autónoma de Ciudad Juárez, Anillo Envolvente Pronaf y Estocolmo s/n, C.P. 32310 Cd. Juárez, Chih, Mexico; 3 Centro de Investigación en Biotecnología Aplicada, Instituto Politécnico Nacional, Tepetitla de Lardizabal, Tlaxcala, Doctorado en Biotecnología, Red de Investigación en Biotecnología IPN, México, Mexico; 4 Centro de Investigación en Ciencia Aplicada y Tecnología Avanzada, Unidad Legaria, Instituto Politécnico Nacional, Calzada Legaria No. 694 Col. Irrigación, Delg. Miguel Hidalgo, Mexico, C.P. 11500 D.F. Mexico

**Keywords:** Essential oil, Breast cancer, Cytotoxic

## Abstract

**Background:**

*Decatropis bicolor* (Zucc.)Radlk is a plant that has been traditionally used for the treatment of breast cancer in some communities of Mexico. So, the aim of this study was to determine the cytotoxic and apoptotic effect of the essential oil of *Decatropis bicolor* against breast cancer cell line, MDA-MB-231.

**Methods:**

The essential oil obtained from hydrodestillation of leaves of *Decatropis bicolor* was studied for its biological activity against breast cancer cells MDA-MB-231 by MTT assay, Hematoxylin-eosin stain, Annexin V-FITC, TUNEL and western blot assays and for its chemical composition by GC-MS.

**Results:**

The results showed a relevant cytotoxic effect of the essential oil towards MDA-MB-231 cells in a dose- and time- dependent manner, with an IC_50_ of 53.81 ± 1.691 μg/ml but not in the epithelial mammary cell line MCF10A (207.51 ± 3.26 μg/ml). Morphological examination displayed apoptotic characteristics in the treated cells like cell size reduction, membrane blebbing and apoptotic bodies. In addition, the apoptotic rate significantly increased as well as DNA fragmentation and western blot analysis revealed that the essential oil induced apoptosis in the MDA-MB-231 cells via intrinsic pathways due to the activation of Bax, caspases 9 and 3. Phytochemical analysis of the *Decatropis bicolor* essential oil showed the presence of twenty-three compounds. Major components of the oil were 1,5-cyclooctadiene,3-(methyl-2)propenyl (18.38 %), β-terpineol (8.16 %) and 1-(3-methyl-cyclopent-2-enyl)-cyclohexene (6.12 %).

**Conclusions:**

This study suggests that essential oil of *Decatropis bicolor* has a potential cytotoxic and antitumoral effect against breast cancer cells, with the presence of potential bioactive compounds. Our results contribute to the validation of the anticancer activity of the plant in Mexican traditional medicine.

**Electronic supplementary material:**

The online version of this article (doi:10.1186/s12906-016-1136-7) contains supplementary material, which is available to authorized users.

## Background

Cancer is a leading cause of mortality worldwide, and according to the American Cancer Society, deaths from cancer constitute 2–3 % of the annual deaths recorded worldwide. An estimated 14.1 million new cancer cases occurred in 2012, and breast cancer was the most common cancer diagnosed in women, representing 25.2 % of all new cases in women [1]. Chemotherapy is a major treatment modality that is used for the control of breast cancer; however, the drugs used exhibit severe toxicity to normal tissues, causing serious side effects [2]. Therefore, many cancer patients seek alternative and/or complementary methods of treatment, such as medicinal plants [3], and there is an urgent need for novel treatment options with improved properties.

Plants are a potential source for drug discovery and the development of cancer chemoprevention. In fact, at least 60 % of the currently used anticancer agents, such as paclitaxel or the vinca alkaloids, vincristine and vinblastine, are derived from natural sources [4]. Several reports indicate that the anticancer activity of medicinal plants is due to the presence of different metabolites. Medicinal plants possess no toxicity compared to modern (allopathic) drugs [4, 5]. The study of new natural products with anticancer activity is important to synthesize new chemical derivatives based on bioactivity and the mechanism of action [5]. Additionally, the essential oils obtained from natural sources have become increasingly popular as naturally occurring bioactive agents [6]. Essential oils (EOs) are volatile complex compounds characterized by a strong odor that are formed by aromatic plants as secondary metabolites. They have been widely used for bactericidal, insecticidal, fungicidal, antioxidant, anticancer, cardiovascular, cosmetic and food applications [7, 8]. EOs obtained from medicinal plants have biological effects via the induction of apoptosis in various cancer cell lines and are promising for the development of novel anticancer agents.

Rutaceae is a plant family which comprises about 161 genera and 1813 species distributed worldwide [9]. Some plants of this family have been widely used in traditional medicine and in the cosmetic, food and pharmaceutical industries, where both, the extracts and the EOs have been proven [10–12]. In particularly, Mexico has a traditional of using medicinal plants from Rutaceae family such as *Decatropis bicolor* (Zucc.)Radlk. This plant is commonly known as arantho, is a 2–3-m tall shrub with small white flowers that is distributed from Mexico to Centroamerica. Several studies demonstrated antifungal [13] and anti-inflammatory activities of different extracts of this plant [14]. The aerial parts of *D. bicolor* are traditionally used for ailments, such as backache, headache, flu, some injuries, and cancer. In communities such as El Cardonal, in Hidalgo State, Mexico, the leaves of *D. bicolor* are used to prepare infusions with approximately 5 g of aerial parts per 1 lt of water, boiled for 15 min and drunk as daily water for the treatment of breast cancer [15–18]; therefore, evaluating the effects of the extracts and the EO of this plant is important to determine its antitumoral activity. Moreover, the plant is used to treat certain inflammatory and oxidative diseases and may have anticancer effects because there is a relationship between the production of reactive oxygen species and the origin of oxidation and inflammation that can lead to cancer.

The objective of the present study was to assess the cytotoxic activity of different plant extracts and the EO of arantho in the metastatic breast cancer cell line, MDA-MB-231, to determine their specific anticancer activities using various assays.

## Methods

### Plant material and extraction

The plant was collected in El Cardonal, Hidalgo State, Mexico on April, 2013. Taxonomic identification of the plant was performed by a botanist at the herbarium Izta of the FES-Iztacala, UNAM (Universidad Nacional Autonoma de Mexico), and a voucher specimen (1917) was deposited in the herbarium.

To prepare the different extracts, maceration technique was used, the leaves were washed and dried at room temperature and then ground into a powder. Four different solvents, water, ethanol, acetone, and hexane, were used. For each extraction, 10 g of the plant was dissolved in 100 mL of the different solvents and left it to macerate in the dark for 24 h. Then, each extract was filtered and either lyophilized (water) or vacuum-evaporated (ethanol, acetone, hexane). For EO isolation, fresh leaves (1 kg) were chopped and hydrodistilled separately for 4 h using a low pressure and low temperature method reported previously [19]. Leaves were ground with water in a blender, deposited into a flask and then brought to a boil. The vapors were condensed on a cold surface using a condenser. The EO was separated based on the difference in density and immiscibility, which was then collected and stored at 4 ° C until use. Each extract and the EO were dissolved in 0.1 % dimethylsulfoxide (DMSO) and then diluted with DMEM to the desired final concentration.

### EO analysis by the gas chromatography–mass spectrometry (GC-MS) method

EO was diluted in dichloromethane at a ratio of 2:48. A volume of 1 μL was manually injected in the split mode into a GC–MS (Perkin Elmer, Turbo Mass Autosystem XL, (Norwalk, CT)) equipped with an HP-FFAP capillary column 19091 F-413 (30 m*0.32 id*0.25 μm film thickness). The injection port was at 180 °C, and the oven temperature was set at 50 °C, increased to 130 °C at a rate of 6 °C min^−1^ and maintained for 3 min. A second set was used at 200 °C, with an increase of 8 °C min^−1^ over 8 min. The carrier gas was high-purity helium at 8 psi. The selective mass detector was a quadrupole Perkin Elmer TurboMass with an electronic impact ionization system at 70 eV and at 215 °C. The results are presented as the relative area % of the total-ion chromatogram, and the percentage composition was calculated by the normalization method of peak areas from the GC as the average value of three injections of oil without correction factors. The compounds were identified by comparing the spectra of the compounds from the EO with the spectral database (NIST MS Search 1.7). The linear retention indices (RI) of the volatile compounds were calculated with *n*-alkanes series (C_10_-C_26_).

### Cell culture

The breast adenocarcinoma MDA-MB-231 and the epithelial mammary cell line MCF10-A were purchased from American Type Culture Collection (ATCC, Rockville). MDA-MB-231 cell line was maintained in DMEM (Dulbecco’s Modified Eagle Medium, Gibco) supplemented with 5 % fetal bovine serum (Invitrogen). MCF10-A cell line was maintained in DMEM/F12 medium supplemented with 10 % serum fetal bovine, hydrocortisone (1 mg/mL), EGF (100 mg/mL) and insulin (100 mg/mL). The cells were grown at 37 °C in a humidified atmosphere of 5 % CO_2_.

### MTT viability assay

Cell viability was evaluated by a MTT (3-(4, 5- dimethylthiazol-2-yl)-2, 5-diphenyl tetrazolium bromide) assay using a modified method of Mosmann [20]. Briefly, 7000 cells were seeded in 96 microplates and incubated for 24 h at 37 °C in 5 % CO_2_. Next, the medium was removed and replaced with fresh medium with or without treatment. The cells were treated using different concentrations of each extract (50–400 μg/mL) and the EO (20–100 μg/mL) and were incubated for 24, 48 and 72 h. Paclitaxel (0.25 μg/mL) was used as the positive control, and cells with medium alone and 0.1 % DMSO were used as negative controls. After incubation, 20 μl of MTT solution (5 mg/mL) was added to each well and incubated for 3 h. Then, the medium containing MTT was replaced with 100 μl of DMSO and the absorbance was measured at 570 nm with an ELISA reader (Labsystem Multiskan Ms). Each experiment was performed in triplicate and repeated three times. Cell viability was expressed as the percentage of control cells. The IC_50_ (50 % inhibitory concentration) was calculated using GraphPad Prism 5.0 software.

### Cell morphology analysis

EO exhibited the greatest cytotoxic effect on MDA-MB-231 breast cancer cells; therefore, the following assays were performed to analyze the apoptotic effect of the EO on this cell line. To analyze the morphological changes in the cells caused by EO, the cells were stained with hematoxylin and eosin (H&E) technique. MDA-MB-231 cells (1x10^4^) were seeded on a 6-well plate for 24 h. After incubation and attachment, the cells were treated with EO using the IC_50_ concentration (53.81 ± 1.691 μg/mL) for 3, 6, 12 and 24 h. Then, the cells were washed with PBS pH 7.4 and fixed in a 4 % paraformaldehyde solution for 30 min. The cells were washed again and stained with H&E methods and observed under a light microscope (Nikon Eclipse TE300).

### Annexin V-FITC binding assay

Apoptosis was determined using the Annexin V-FITC Apoptosis Detection kit (Biovision) according to the manufacturer’s instructions. Briefly, MDA-MB-231 cells were cultured at a density of 5x10^5^ and allowed to attach overnight, followed by treatment with the EO (IC_50_ value) or the controls for 0.5, 1.5, 3, 6, 12 and 24 h. The cells were then harvested by trypsinization, centrifuged and washed with PBS and incubated in binding buffer with 5 μl of annexin-V and 5 μl of propidium iodide for 5 min in the dark at room temperature.

Analysis was performed by flow cytometry (FACScan, Becton Dickinson Cytometer). A minimum of 10,000 events was collected for analysis.

### Terminal deoxynucleotidyl transferase-mediated dUTP nick end labeling (TUNEL)

DNA fragmentation, a characteristic of apoptosis, was assessed using an In Situ Cell Death Detection Kit AP (Roche) following the manufacturer’s instructions. Briefly, breast cancer cells (5x10^4^) were cultured on 6-well plates over coverslips and incubated for 24 h. The cells were then treated with EO (IC_50_ value) for different times (1.5, 3, 6, 12 and 24 h). Also cells were treated with media alone, DNAse (3000 U/mL) or paclitaxel (0.25 μg/mL) for 24 h. Next, the cells were washed with PBS and fixed in paraformaldehyde solution. The cells were incubated in a permeabilization solution (0.1 % Triton X-100 in 0.1 % sodium citrate) for 1 h at 4 ° C and then, the cells were treated with solution A and B for 1 h in the dark at 37 ° C. Analysis was performed by fluorescence microscopy (Nikon diaphot 200) with a laser scanning confocal imaging system (MCRR 1024).

### Western blotting analysis

The analysis of protein expression involved in the two main pathways of apoptosis was performed by western blot. MDA-MB-231 breast cancer cells were seeded (5 x 10^5^) for 24 h, and the cells were then treated with EO (IC_50_ value) or controls for 0.5, 1.5, 3, 6, 12 and 24 h. The cells were harvested by trypsinization and washed with PBS, and the pellet was resuspended in 20 μl of a protease inhibitor cocktail solution and 100 μl of cold lysis buffer (50 mM Tris–HCl, 150 mM NaCl, 0.1 % SDS, 1 % NP-40, 1 mmol phenylmethylsulfonyl fluoride, 100 μM leupeptin, and 2 μg/L aprotinin). The protein lysates were centrifuged at 10, 000 rpm for 10 min at 4 °C. The protein concentration was determined by Bradford assay. Protein extracts were reconstituted in sample buffer and the mixture was boiled for 5 min. Equal amounts (30 μg) of the denatured proteins were loaded into each lane and separated on a 15 % SDS polyacrylamide gel, followed by the transfer of the proteins to a 0.45-μm nitrocellulose membranes for 2 h. The membranes were blocked with 5 % non-fat dry milk and then incubated with primary rabbit polyclonal antibodies to procaspase-3 (1:200, Santa Cruz, CA), procaspase-8 (1:200, Santa Cruz, CA), procaspase-9 (1:200, Santa Cruz, CA), Bax (1:200, Santa Cruz, CA), Bcl-2 (1:200, Santa Cruz, CA), PCNA (1:100, Santa Cruz, CA), or β-actin (1:1000, Santa Cruz, CA) as a positive control for 2 h at 4 ° C. They were then incubated with horseradish peroxidase-conjugated goat anti-rabbit or anti-mouse secondary antibody (1:2000) for 2 h before being visualized with diaminobenzidine solution. All experiments were performed in triplicate.

### Statistical analysis

All data are expressed as means ± S.E. One-way ANOVA followed by Tukey test was used to compare all groups to each other. For all tests, *p* < 0.05 was considered significant.

## Results

### Effect of the different extracts and EO of arantho on viability

**Fig. 1:**
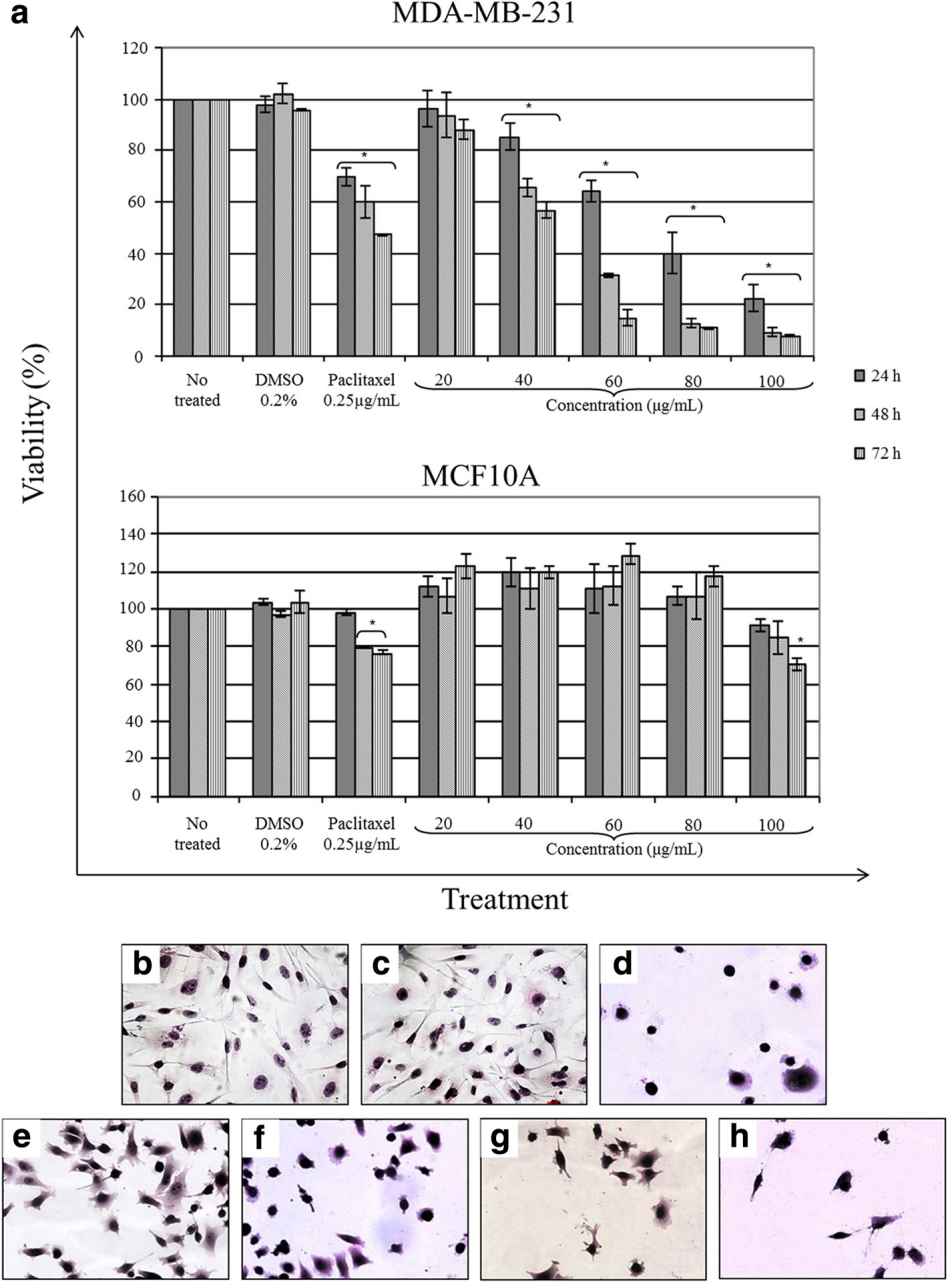
Effect of arantho EO on the cellular viability and on morphological changes **a** Cell viability was evaluated by MTT assays using different concentrations and times of incubation (24, 48 and 72 h) of EO on MDA-MB-231, a breast carcinoma cell line, and MCF-10A, an epithelial breast cell line. **b** Morphological analysis of MDA-MB-231 breast cancer cells without treatment. **c** Cells incubated with 0.2 % DMSO. **d** Cells exposed to paclitaxel (0.25 μg/mL). **e**-**h** Cells incubated with EO (53.81 μg/mL) for 3, 6, 12 and 24 h, respectively. H&E staining, 40x

The cytotoxic activities of the extracts and EO from arantho were analyzed by MTT assays performed on the MDA-MB-231 breast cancer cell line. The aqueous extract did not show any cytotoxic activity at any concentration or treatment period against these cancer cells. However, the ethanolic, acetonic and hexanic extracts showed a cytotoxic effect in a time- and concentration-dependent manner, with IC_50_ values of 128.20 ± 2.035, 203.2 ± 2.3 and 450.7 ± 2.657 μg/mL, respectively (Additional file 1). As shown in Fig. 1a, a better cytotoxic effect was observed when the MDA-MB-231 cells were exposed to the EO. After 24 h of incubation, the cell viability decreased 15, 36, 60 and 77 % with 40, 60, 80 and 100 μg/mL, respectively. In addition, after 48 h of treatment, the cell viability decreased 35, 69, 88 and 91 % using doses of 40, 60, 80 and 100 μg/mL, respectively. A greater cytotoxic effect was observed after 72 h of incubation, with a reduction of cell viability of more than 85 % for 60, 80 and 100 μg/mL. The cytotoxic effect of the EO was dose- and time- dependent, with an IC_50_ of 53.81 ± 1.691 μg/mL. Paclitaxel (0.25 μg/mL) was used as a positive control and also caused a decrease in viability of 30, 40 and 53 % at 24, 48 and 72 h, respectively, whereas MDA-MB-231 cells without treatment maintained a viability of 100 %. These results demonstrate an important and significant cytotoxic effect of the arantho EO against MDA-MB-231 cells.

To analyze the specificity of the EO against breast cancer cells, an epithelial breast cell line, MCF-10A, was used. The results showed that MCF-10A cells treated with 20 to 80 μg/mL EO maintained cell viability similar to the negative controls (100 %) during all treatments. Higher doses (100 μg/mL) given to the normal epithelial cells for 72 h decreased the cell viability 25 % (Fig. 1a). The IC_50_ value for normal epithelial cells was 207.51 ± 3.26 μg/mL, representing a 2.84-fold higher concentration compared to the IC_50_ concentration for breast cancer cells. These results confirmed that EO was selective against breast cancer cells.

### Morphological changes on MDA-MB-231 cells exposed to arantho EO

To determine whether the reduced cell viability of the cancer cells resulted from cell death induction, MDA-MB-231 cells were incubated with the EO using the IC_50_ value for different times and were stained with H&E. Non-treated cells possessed an elongated shape; a centrally located nucleus, forming a confluent colony; and were completely attached to the plate (Fig. 1b, c). By contrast, cells exposed to EO showed relevant morphological changes after 3 h of incubation, including cell size reduction, membrane blebbing, cell shrinkage and loss of colony formation (Fig. 1e). These changes increased with time, and after 6, 12 and 24 h of incubation, the cells appeared to be rounded and apoptotic bodies were present (Fig. 1f-h). Similar characteristics were observed in breast cancer cells exposed to paclitaxel (Fig. 1d). These morphological characteristics suggest the induction of apoptosis by arantho EO.

### Apoptotic induction by the arantho EO

**Fig. 2:**
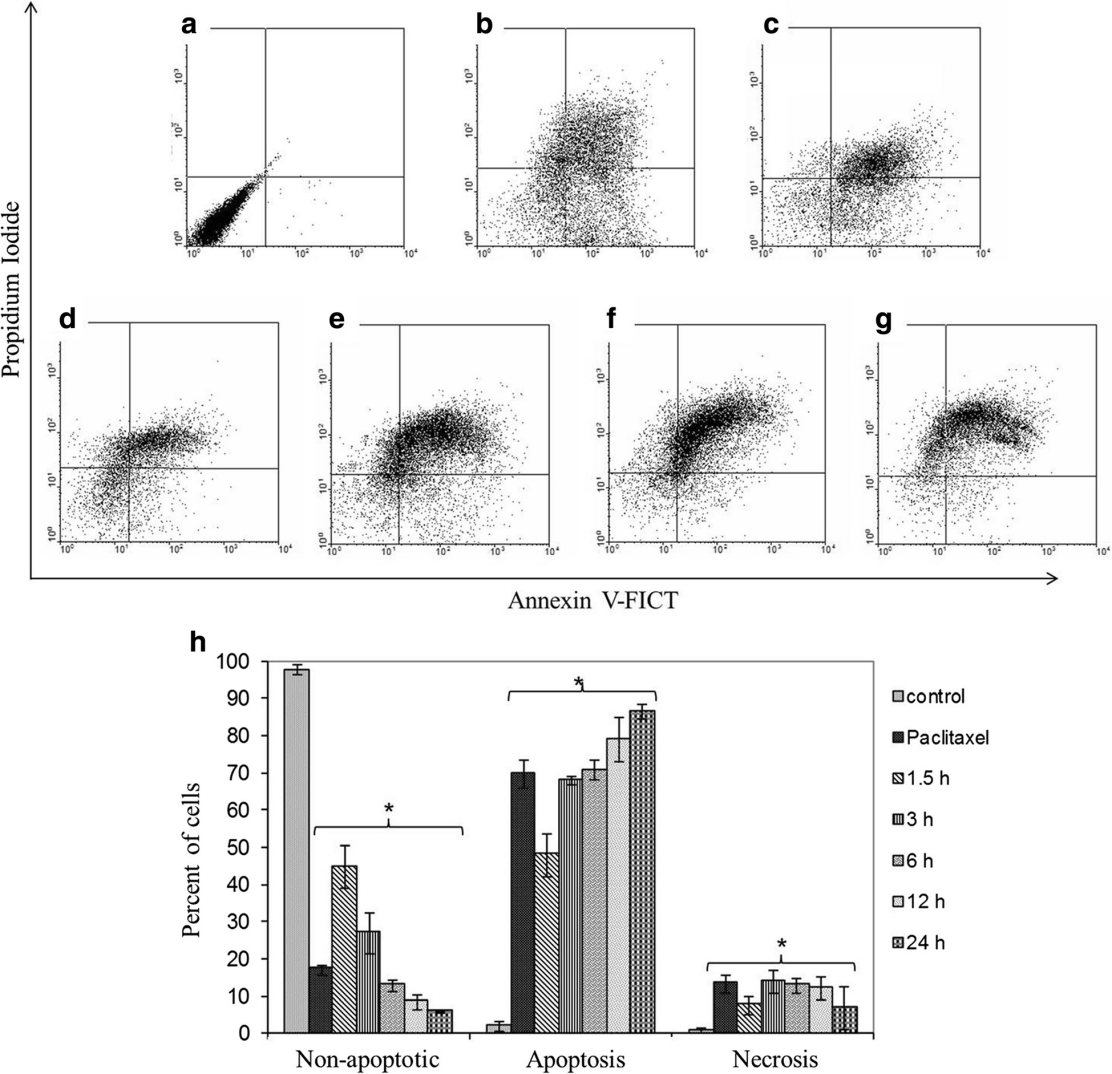
Apoptotic induction of the arantho EO on MDA-MB-231 breast cancer cells analyzed by Annexin V-FITC assays. The results were analyzed by flow cytometry. Cancer cells with **a** no treatment; **b** treatment with 0.25 μg/mL paclitaxel; and **c**-**g** treatment with EO (53.81 μg/mL) for 1.5, 3, 6, 12 and 24 h, respectively. **h** Percent of live, apoptotic and necrotic breast cancer cells with the different treatments. Graphs represent the average percentages of apoptosis of three independent experiments

To determine and quantify cell death, MDA-MB-231 cells were incubated with EO (53.81 μg/mL) and analyzed by Annexin V-FITC assays. Control cells without treatment showed 98 % non-apoptotic live cells (Fig. 2a, h) and a very low percent (1–2 %) of apoptosis and necrosis. By contrast, cells incubated with 0.25 μg/mL paclitaxel showed only 18 % non-apoptotic live cells and displayed 69 and 12 % apoptotic and necrotic cells, respectively (Fig. 2b, h). Cancer cells exposed to 53.81 μg/mL of EO showed decreased non-apoptotic live cells (from 45 to 5 % after 1.5 and 24 h, respectively) and increased apoptotic cells (from 47 to 85 % after 1.5 and 24 h, respectively) in a time-dependent manner (Fig. 2c-h). The percent of necrotic cells was similar for the different treatments. These results indicate that EO induces apoptosis rather than necrosis in MDA-MB-231 breast cancer cells, and this is consistent with the morphological changes observed by H&E staining.

### Arantho EO induced DNA fragmentation in MDA-MB-231 cells

**Fig. 3:**
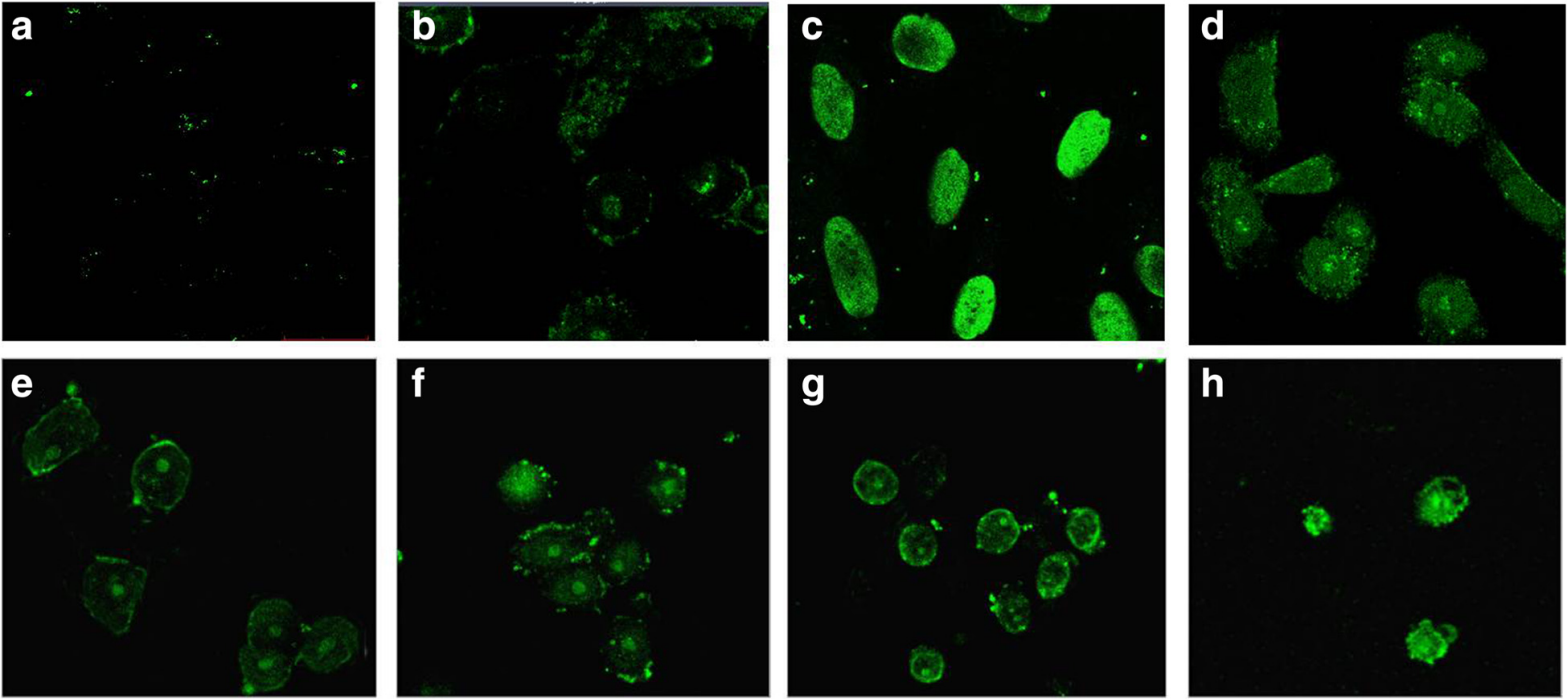
Analysis of DNA fragmentation by TUNEL assay in MDA-MB-231 breast cancer cells incubated with arantho EO. **a** Cells without treatment. **b** Cells exposed to 0.25 μg/mL paclitaxel. **c** Cells treated with DNAse (3000 U/mL). **d**-**h** Cells incubated with 53.81 μg/mL of EO for 1.5, 3, 6, 12 and 24 h, respectively

To determine the ability of the arantho EO to trigger DNA fragmentation as a feature of apoptosis, TUNEL assays were performed. Breast cancer cells treated with EO (53.81 μg/mL) showed an increased in DNA fragmentation after 1.5, 3, 6, 12 and 24 h of incubation (Fig. 3d, e, f, g and h, respectively). As expected, cancer cells exposed to paclitaxel and DNase were also positively stained in the assay (Fig. 3b and c, respectively). As a control, cancer cells without treatment did not exhibit fluorescence staining in the nucleus (Fig. 3a). These findings indicate that arantho EO induced cell death in MDA-MB-231 cells by apoptosis rather than necrosis.

### Apoptotic effect of arantho EO on MDA-MB-231 cells is mediated by activation of caspase cascades

**Fig. 4:**
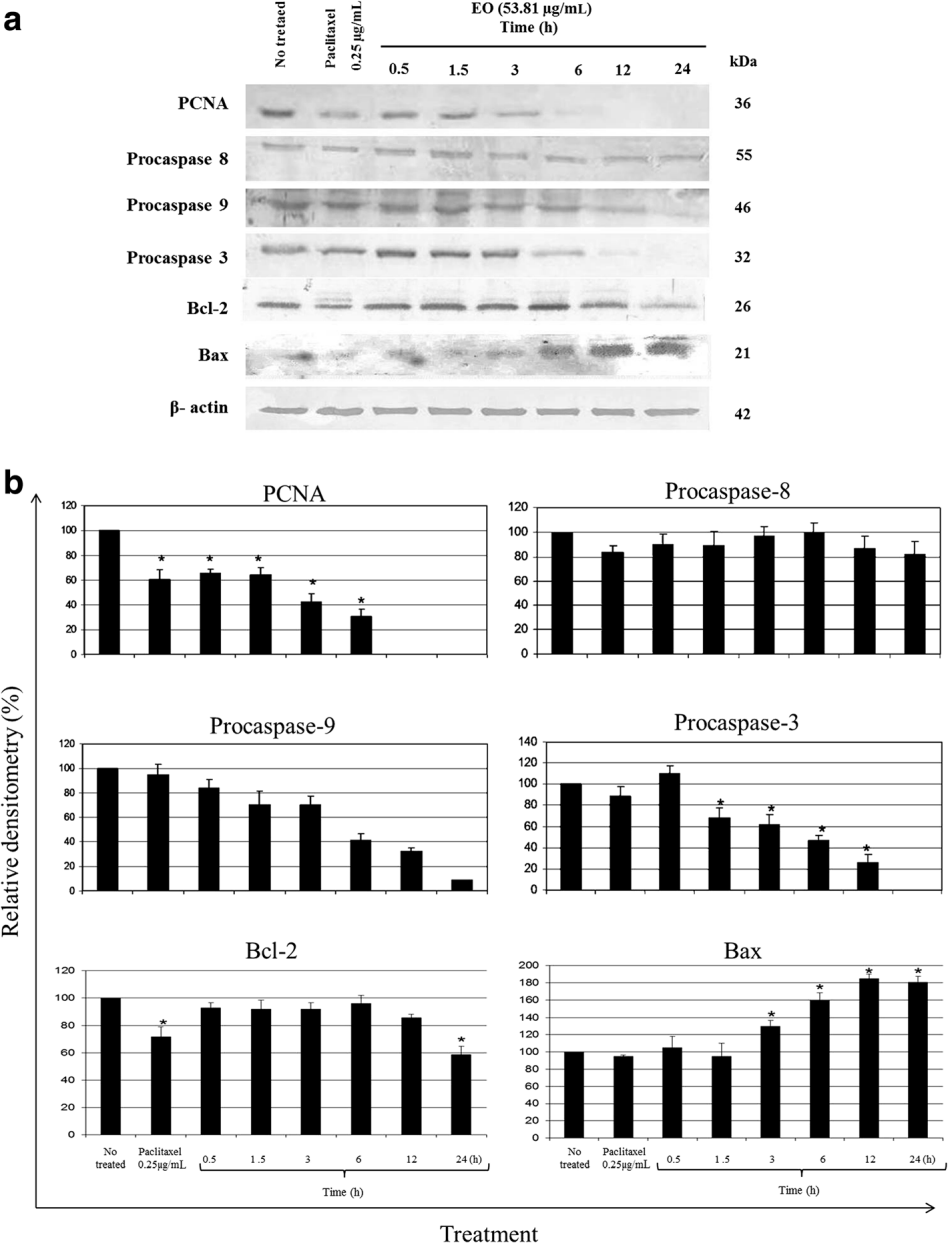
Western blot analysis of MDA-MB-231 breast cancer cells incubated with arantho EO (53.81 μg/mL) for 0.5, 1.5, 3, 6, 12 and 24 h. **a** Analysis of the expression of PCNA; procaspase-3, 8 and 9; Bcl-2; and Bax. The membranes were also detected with an antibody against β-actin as a loading control. **b** Relative densitometry of each protein

To analyze the activation of an apoptotic pathway, western blot assays were performed to evaluate the expression of proteins, such as PCNA (proliferating cell nuclear antigen); procaspases 3, 8 and 9; Bcl-2; Bax; and β-actin. As shown in Fig. 4a and b, the expression of the PCNA protein decreased by 35 % after 30 min of incubation with EO. In addition, at 12 and 24 h, expression was not detected compared to the untreated cells. By contrast, the expression of procaspase-8 (55 kDa protein inactive form) was constant for the different treatments, suggesting that the extrinsic pathway was not activated. However, some changes were observed in the expression of procaspase-9 and 3, corresponding to the inactive form of the protein for each. The expression of procaspase-9 decreased from 30 to 90 % after 1.5 to 24 h of incubation. The expression of procaspase-3 decreased by 30 % at 1.5 h of incubation, but at 24 h, the protein was not detected. Bcl-2 is an anti-apoptotic protein. It decreased after 12 and 24 h (18 and 40 %, respectively) of incubation with the breast cancer cells exposed to EO. Bax, a proapoptotic protein, increased markedly after 3 h of incubation (25 %). β-actin was used as the loading control and showed equal intensity bands in all samples. These results indicated that EO induced apoptotic cell death via activation of the intrinsic pathway.

### Chemical composition of Arantho EO

EO was obtained using the hydrodistillation method from the fresh leaves of arantho with a yield of 23 %. GC-MS analyses were then used to determine the composition of the extract. Twenty-three compounds, representing 76.56 % of the EO, were identified (Table 1). These components were separated into seven classes, hydrocarbons (24.50 %), terpenes (17.85 %), alcohols (17.75 %), carboxylic acids (12.33 %), aldehydes (3.04 %), benzene derivatives (1.07 %) and others (23.46 %). The major components of the oil were 1,5-cyclooctadiene,3-(methyl-2) propenyl (18.38 %), β-terpineol (8.16 %), 1-(3-methyl-cyclopent-2-enyl)-cyclohexene (6.12 %), 2-heptanol acetate (5.13 %), and limonene (4.49 %). All other components were present at less than 4 % abundance (Table 1).

**Table 1:** EO composition of arantho leaves

No	Compound	Retention time (min)	KI	Abundance (%)
1	α-Pinene	1.8	885	2.60
2	Camphene	2.06	1008	0.91
3	β-Pinene	2.37	1042	1.18
4	β-Phellandrene	2.5	1057	1.03
5	β-Myrcene	2.92	1104	1.05
6	Limonene	3.34	1151	4.49
7	2-Hexenal	3.63	1183	3.04
8	3-Carene	3.97	1211	0.94
9	2-Heptanol acetate	4.29	1230	5.13
10	2-Heptanol	4.35	1233	3.20
11	Acetic acid, octyl ester	6.3	1348	1.28
12	Eucalyptol	6.90	1383	1.10
13	Linalool	7.2	1401	1.53
14	1-(3-Methyl-cyclopent-2-enyl)-cyclohexene	7.38	1410	6.12
15	Acetic acid,1,7.7-trime-bicyclo [2.2.1]	7.51	1417	3.82
	hept-2-yl ester			
16	Terpineol	7.65	1424	1.04
17	Acetic acid, decyl ester	8.36	1461	2.11
18	β-Terpineol	8.91	1490	8.16
19	1,5-Cyclooctadiene,3-(methyl-2)propenyl	9.45	1518	18.38
20	Germacrene	12.16	1657	3.07
21	Methyl salicylate	13.5	1684	1.07
22	Elemol	16.71	1870	1.07
23	Hedycaryol	19.36	1980	1.05

*KI* Kovats Retention Index

## Discussion

*D. bicolor* is a widely used plant by the people of several communities of central Mexico for treating breast cancer. However, little is known regarding its anti-proliferative effects in this disease. Therefore, the purpose of this study was to determine whether *D. bicolor*, commonly known as arantho, has a cytotoxic effect against breast cancer cells. In the present study, we obtained different extracts, as well as the EO of this plant, to investigate its anti-proliferative effect and to characterize the type of cell death induced.

Several studies indicate that EOs possess chemo-preventive potential, anti-tumoral activity and it function to induce apoptosis in different cancer cell lines [12, 21]. In particularly, plants of the Rutaceae family are characterized by the presence of odoriferous glands containing aromatic essences, and their EOs are cytotoxic in different models of cancer. These plants include *Citrus sp.* [11, 22] *Zanthopylum fagara* [23] and *Ruta graveolens* [24]. Several metabolites are representative of this family; however, the different phytochemical profiles are important for their biological activity.

In the present study, we showed that arantho EO, which belongs to the Rutaceae family, had time- and concentration-selective cytotoxic activity against MDA-MB-231, a highly invasive estrogen receptor-negative breast cancer cell line, with an IC_50_ value of 53.81 μg/mL, and possessed better effects than the different extracts of *D. bicolor*, while no effect was observed using aqueous extract. The type of compounds extracted from the plant depends on the polarity, acidity and alkalinity of the solvent; which means that polar solvents such as water tends to extract polar compounds (alcohols, amines, acids, esters, sugars, glycosides), while low- or non-polar solvents such as the ethanolic, acetonic, hexanic or EO tends to extract different kinds or different quantities of non-polar compounds (fats, waxes, volatile oils) [25]. It is also possible that concentrations of active constituents in the extracts and in the EO may vary depending on the extraction conditions; the extraction time; the temperature or the extraction method. Thus, results suggest that differential content and/or concentrations specially of polar compounds, extracted with each extract influenced in the cytotoxicity observed [26].

On the other hand, the cytotoxicity was not observed in the MCF-10A epithelial mammary cell line when low concentrations of EO were used. The differential EO effect between these cell lines may be due to the specific genetic profile; to the membrane potential derived from specific ion channels; to the distinct adhesion molecules in the surface, or to other characteristics of breast cancer cells making it more sensitive to EO [27].

To determine whether the cytotoxic activity of EO was due to apoptosis, MDA-MB-231 cells were treated for 0 – 24 h with the IC_50_ value of arantho and analyzed by H&E staining and TUNEL assays. Apoptosis is programmed cell death that maintains cellular homeostasis between cell division and cell death [28]. This physiological process induces cellular self-destruction, generating diverse morphological and biochemical features in the nucleus and cytoplasm. Apoptosis is a primary death induced by natural and certain synthetic compounds with antitumoral activities [28, 29]. Interestingly, arantho EO induced early (3 h) cell detachment, floating cell size reduction, membrane blebbing, and cell shrinkage. However, it also induced apoptotic body formation and chromatin condensation, all of which are typical features of apoptosis [29]. Moreover, DNA fragmentation was observed earlier than 3 h (1.5 h) of exposure to EO, and increased in a time-dependent manner to 24 h, indicating that the compound or compounds induce cell damage almost immediately, rather than the metabolites. This was confirmed when we performed a quantitative analysis of apoptosis using Annexin V-FITC because the results revealed that 47 % of the MDA-MB-231 cells exposed to EO for 1.5 h are in apoptosis, and at 24 h, the apoptosis rate was 85 %. Furthermore, necrosis was no greater than 18 % in all of the tested conditions. Taken together, these findings indicate that the EO of *D. bicolor* induced cell death in MDA-MB-231 cells due to apoptotic rather than necrotic effects. This is consistent with the goal of potential anticancer drugs, in which a primary characteristic is to induce apoptosis. Different studies using EOs of medicinal plants have demonstrated their potential anti-cancer activity by inducing apoptosis or cell cycle arrest [30].

Apoptosis is activated by two different pathways, the intrinsic and extrinsic pathways. The extrinsic pathway is mediated by death receptors, whereas the intrinsic or mitochondrial pathway is triggered by the release of apoptogenic proteins, such as cytochrome c, which activate caspase proteins that are the main effector molecules that induce this process [28]. Arantho EO activated caspases 9 and 3 at short treatment times. Caspase 3 is an executer protein that cleaves different substrates to generate DNA fragmentation and morphological changes, such as the appearance of apoptotic bodies. These characteristics were observed at different times of exposure. By contrast, caspase 8, a protein that self-cleaves as a result of the activation of the extrinsic pathway, maintained expression when breast cancer cells were exposed to arantho EO. This result indicated that the extrinsic pathway was not activated. Additionally, the EOs of *Citrus bergamia* (0.005 %) and *Aniba rosaeodora* (400 nL/mL) also induce apoptosis via the activation of caspase 3 and 9 after 1 and 2 h of treatment for the SH-SY5Y and A431 cell lines, respectively [12]. By contrast, the Bcl-2 family is also a key regulator of apoptosis, and it includes apoptotic (Bax, Bak, and Bid) and antiapoptotic molecules (Bcl-2 and Bcl-xl) [31]. In our study, the expression of Bax was increased in breast cancer cells exposed to arantho EO as early as 3 h. These data were consistent with the expression of proteins involved in the intrinsic pathway, and it was expected that the expression of Bcl-2 decreased in the treated cancer cells. Therefore, the results are consistent with other EOs that downregulate Bcl-2 protein expression in different cancer cells to induce apoptosis as a relevant strategy to control cancer development and progression [30]. These observations suggest that EO is an interesting natural antitumoral candidate for the treatment of cancer. We propose that EO activates the Bax protein, which permeabilizes the outer membrane of mitochondria. This generates large pores that allow the release of cytochrome c and activate caspase 9 and caspase 3, resulting in cell death.

The cytotoxic activities of natural products, such as EOs, are mainly attributed to the presence of different bioactive compounds, including terpenes, terpenoids, alkanes, and aromatic components [32]. Interestingly, phytochemical analysis of the *D. bicolor* EO showed the presence of different compounds, particularly hydrocarbons and terpenes. This result is consistent with the consensus of the chemical composition of EOs, indicating that the three main groups of compounds are terpenes and terpenoids, aromatics (phenolic) and aliphatics [6]. However, 1-5-cyclooctadiene,3-(methyl-2)propenyl and 1-(3-methyl-cyclopent-2-enyl-cyclohexene, two hydrocarbons that were major compounds identified in *D. bicolor* EO, may be responsible for the cytotoxic activity of this EO towards the MDA-MB-231 breast cancer cells, although nothing is known of its antitumoral activity. Nevertheless, other constituents of EO may contribute to the cytotoxicity. EOs isolated from antitumoral plants of the Rutaceae family of the genus *Citrus* (*C. aurantium, C. limon* and *C. reticulata*) possess a highly different chemical composition compared to *D. bicolor*, which may be due to the difference in their genetic profile and environmental conditions [10]. Moreover, several of the metabolites identified were isolated in other studies and demonstrated cytotoxic and antitumoral activities. One of the three major compounds identified in *D. bicolor* EO is α-terpineol, which decreased both the viability of the MCF-7 breast cancer cell with an IC_50_ value of 16.2 μM [33] and the expression of NF-kB [34]. Other important less abundant compounds in *D. bicolor* EO that have been isolated from other medicinal plants and possess antitumoral effect are limonene, α-pinene and linalool. α-Pinene is a monoterpene that induces apoptosis in SK-OV3, HO-8910, Bel-7402 [21] and C32 cancer cells [35]. Linalool had a cytotoxic effect on melanoma [35], breast cancer cells [36] and hepatocarcinoma cells [37]. Limonene is a monoterpene representative of the Rutaceae family. It inhibits the growth of different cell lines, such as DU-145 prostate [38], carcinoma cells, with an IC_50_ of 2.8 mM. However, limonene, linalool and α-pinene previously tested on MDA-MB-231 breast cancer cells were not cytotoxic [39]. These investigations indicate that several metabolites present in arantho EO are cytotoxic to different cancer cell lines; however, it is difficult to attribute the biological activity to the entire EO or to one specific compound because the cytostatic effect of an entire plant on cancer cells is often better than the effect of the particular biological active compounds [7]. In addition, due to the lipophilic nature of EO, volatile compounds appear to accumulate in the cell membrane and increase its permeability, resulting in the leakage of enzymes and metabolites that induces cytotoxic and apoptotic effect in cancer cells [6]; however, is necessary to identify the active compound responsible for the cell death of these breast cancer cells.

## Conclusion

In conclusion, several assays demonstrated an important cytotoxic, selective and apoptotic activity of the EO of *D. bicolor* against the breast cancer cell line MDA-MB-231, Additional in vitro and in vivo studies currently under study will allow us to determine the sensitivity of other cell lines and the anti-tumoral potential of the essential oil from arantho (EO).

## Abbreviations

DMEM, Dulbecco’s Modified Eagle Medium; DMSO, dimethylsulfoxide; EO, essential oil; GC-MS, gas chromatography–mass spectrometry; H&E, hematoxylin and eosin; KI, Kovats Retention Index; MTT, 3-(4, 5- dimethylthiazol-2-yl)-2, 5-diphenyl tetrazolium bromide; PCNA, proliferating cell nuclear antigen; TUNEL, terminal deoxynucleotidyl transferase-mediated dUTP nick end labeling.

## Additional file

Additional file 1: MTT assays of the different extracts of *D. bicolor* on MDA-MB-231 breast cancer cell line. The graphics represent the MTT assays of the different extracts (aqueous, ethanolic, acetonic and hexanic) of * D. bicolor *on breast cancer cells MDA-MB-231. The ethanolic, acetonic and hexanic extracts demonstrated a cytotoxic effect in a doses and time dependent manner, obtaining IC_50_ values of 128.20 ± 2.035, 203.2 ± 2.3 and 450.7 ± 2.657 μg/mL, respectively. However, the aqueous extract didn’t showed any cytotoxic activity at any concentration or time. The experiments were performed at least in triplicate and the values are reported as mean ± SE, **P* < 0.05 as compared to control cells (medium alone). Significance was analyzed using One-way ANOVA followed by Tukey test. (PDF 270 kb)
