# Supplementary figures and images for: Decatropis bicolor (Zucc.) Radlk essential oil induces apoptosis of the MDA-MB-231 breast cancer cell line
Source: BMC Complement Altern Med. 2016 Aug 5;16:266. doi: 10.1186/s12906-016-1136-7 (PMC4974778; doi:10.1186/s12906-016-1136-7)

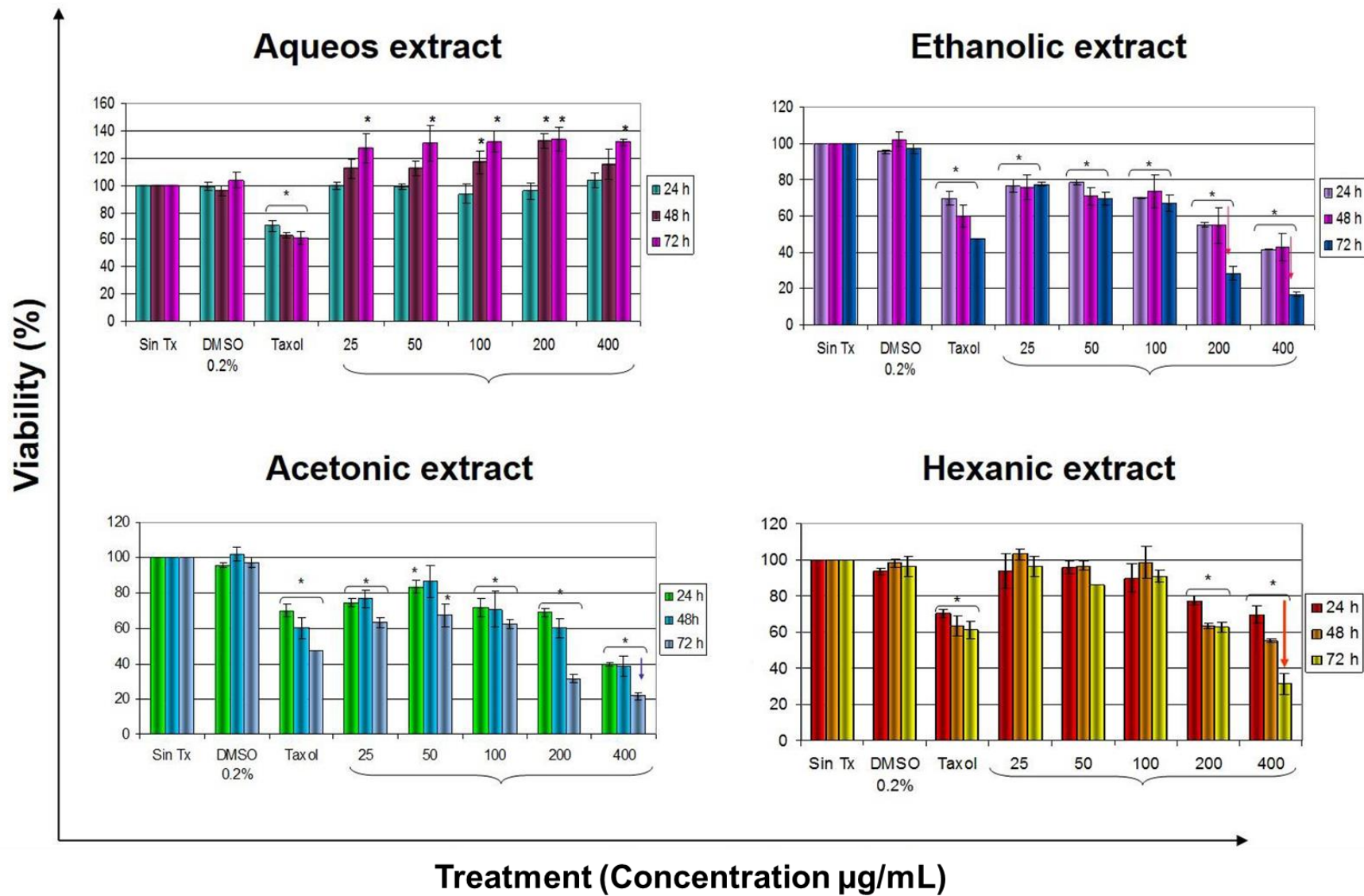

Supplement: Additional file 1: — MTT assays of the different extracts of D. bicolor on MDA-MB-231 breast cancer cell line. The graphics represent the MTT assays of the different extracts (aqueous, ethanolic, acetonic and hexanic) of D. bicolor on breast cancer cells MDA-MB-231. The ethanolic, acetonic and hexanic extracts demonstrated a cytotoxic effect in a doses and time dependent manner, obtaining IC50 values of 128.20 ± 2.035, 203.2 ± 2.3 and 450.7 ± 2.657 μg/mL, respectively. However, the aqueous extract didn’t showed any cytotoxic activity at any concentration or time. The experiments were performed at least in triplicate and the values are reported as mean ± SE, *P < 0.05 as compared to control cells (medium alone). Significance was analyzed using One-way ANOVA followed by Tukey test. (PDF 270 kb) [file 12906_2016_1136_MOESM1_ESM.pdf]
